# Ion conformation and orientational order in a dicationic ionic liquid crystal studied by solid-state nuclear magnetic resonance spectroscopy

**DOI:** 10.1038/s41598-021-85021-y

**Published:** 2021-03-16

**Authors:** Debashis Majhi, Sergey V. Dvinskikh

**Affiliations:** 1 Department of Chemistry, KTH Royal Institute of Technology, 10044 Stockholm, Sweden; 2 School of Chemistry, Tel Aviv University, Ramat Aviv, 6997801 Tel Aviv, Israel; 3 Laboratory of Biomolecular NMR, Saint Petersburg State University, Saint Petersburg, 199034 Russia

**Keywords:** Chemical physics, Liquid crystals, Liquid crystals, Self-assembly, Liquid crystals, Self-assembly, Chemical physics, Solid-state NMR

## Abstract

Ionic liquids crystals belong to a special class of ionic liquids that exhibit thermotropic liquid-crystalline behavior. Recently, dicationic ionic liquid crystals have been reported with a cation containing two single-charged ions covalently linked by a spacer. In ionic liquid crystals, electrostatic and hydrogen bonding interactions in ionic sublayer and van der Waals interaction in hydrophobic domains are the main forces contributing to the mesophase stabilization and determining the molecular orientational order and conformation. How these properties in dicationic materials are compared to those in conventional monocationic analogs? We address this question using a combination of advanced NMR methods and DFT analysis. Dicationic salt 3,3′-(1,6-hexanediyl)bis(1-dodecylimidazolium)dibromide was studied. Local bond order parameters of flexible alkyl side chains, linker chain, and alignment of rigid polar groups were analyzed. The dynamic spacer effectively “decouples” the motion of two ionic moieties. Hence, local order and alignment in dicationic mesophase were similar to those in analogous single-chain monocationic salts. Bond order parameters in the side chains in the dicationic smectic phase were found consistently lower compared to double-chain monocationic analogs, suggesting decreasing contribution of van der Waals forces. Overall dication reorientation in the smectic phase was characterized by low values of orientational order parameter *S*. With increased interaction energy in the polar domain the layered structure is stabilized despite less ordered dications. The results emphasized the trends in the orientational order in ionic liquid crystals and contributed to a better understanding of interparticle interactions driving smectic assembly in this and analogous ionic mesogens.

## Introduction

Ionic liquids crystals (ILCs) belong to a special class of ionic liquids (ILs) with mesogenic functionalities which show thermotropic liquid crystals behaviour^1^. The molecular design of ILCs is commonly based on cations and anions of conventional ionic liquids (ILs) by addition of one or more long alkyl chains^2,3^. The ionic character and rod-shaped structure of cations favor the formation of layered phases^1,2,4^. There are several types such as ammonium, imidazolium, pyridinium based ILCs reported, among these monocationic imidazolium-based salts are the most studied ones^4^. Recently, dicationic ionic liquid crystals (DILCs) have also been presented^5–11^. DILCs are composed of a dication containing two single-charged ions covalently linked by a spacer, and single-charged anions^5^. Increased possibilities for cation and anion combinations, presence of more than one polar and apolar units, and variation in the length and type of spacer group between charged units in dicationic ILCs is beneficial to achieve more variability and tunability of the properties of ionic liquid crystals. Imidazolium-based dicationic ILCs combined with a range of anions have been synthesized and their mesophase behavior has been studied depending on linker type and side-chain length^6–10^. Bipyridinium based DILCs salt have also been reported^11^. Some dicationic ionic liquids (DILs) are more thermally stable than those based on monocationic units^5,12^. Imidazolium based DILCs have found growing applications in areas varying from biochemistry and biology^13–15^ to electrochemistry^16^ and electro-optics^17^ to tribology^18^. Recent studies have demonstrated that dicationic ILs not only show favorable physicochemical properties^19^ but are also significantly different in their dynamic behaviour^20^ as compared to conventional monocationic analogs. Studies of mesophase stability of imidazolium-based DILCs in response to structure modifications have been reported^6–8^. Dicationic ionic liquids with longer pendant alkyl chains are more likely to show the liquids crystal behavior. The mesophase also depends on the nature of cations and changes with the structure of the spacer (linker chain).

In ionic liquid crystals, electrostatic and hydrogen bonding interactions in ionic sublayer and van der Waals forces in hydrophobic domains are the main forces contributing to mesophase stabilization^1^. While Coulomb interaction is ubiquitous in ionic materials, the contribution of the others, more local and directional forces varies dramatically with the structure of ionic units. Hydrogen bond (HB) formation depends on the presence of sufficiently strong both H-bond donors and acceptors. There have been numerous experimental and theoretical studies of H-bonding in nonmesogenic ionic liquids^21,22^. Short-range anisotropic van der Waals forces contribute to the formation of a great variety of complex molecular assemblies in conventional LCs of neutral molecules. Restricted reorientational dynamics of partly aligned anisotropic units in ILCs can also modify the contribution of longer-range electrostatic interactions^23^. The simultaneous presence of polar and apolar regions within the cations induces nano-segregation thereby stabilizing the mesophase. An understanding of the intermolecular interactions in ILCs provides important feedback for the rational selection of ions to design new functional materials for specific applications.

Orientational order parameter *S* is a prime characteristic of LCs. We have previously shown that the *S* value in ILCs greatly varied depending on structural details^24–27^. In single-chain monocationic ILCs, a fragile balance of intermolecular forces led to low *S* values^24–26,28–30^. The degree of orientational ordering has been found to correlate well with physicochemical properties of anions and increased with a phase stability range^25^. On the other hand, an expanded H-bond network in hydrated materials led to higher phase stability with a simultaneous decrease of the order parameter^26^. We have demonstrated that the enhanced contribution of van der Waals interactions in double-chain ILCs led to a greatly increased order parameter^27^. However, the consequence of the larger cation size and anisotropy of the double chain ILCs was difficult to estimate and separate from the effect of the changed balance of the interaction forces. In DILCs with two long alkyl side-chains and a spacer, the rod shape character of the cation is enhanced, similarly to symmetric monocationic ILCs. Hence, an increased contribution of van der Waals forces in apolar domains to mesophase stabilization is expected. On the other hand, the roles of electrostatic and HB forces also increase compared to corresponding monocationic ILCs. By tuning a specific force, while conserving the contribution of other interactions as far as possible, and monitoring induced structural and dynamic changes at a molecular level, one can gain new physical insights into fundamental mechanisms governing ionic mesophase formation.

In the present work, we investigate orientational order, conformational parameters, and alignment of functional molecular groups in a representative DILC material. To the best of our knowledge, no studies of these properties in dicationic ILC materials were reported. We discuss the difference of the conformational and ordering parameters in DILCs and monocationic single and double chain analogs and explain it in terms of a combination of intermolecular forces. A deeper understanding of the relationship between the diverse molecular organization and the physical properties in these materials will facilitate the directed search for cation–anion combinations of ILCs for optimal performance in targeted applications.

As a main experimental method in this study, we apply Solid-State Nuclear Magnetic Resonance (NMR). NMR spectroscopy, supported by molecular simulation, is well-positioned to deliver required atomic- and molecular-level information. NMR techniques based on measuring dipolar spin interactions have been developed for quantitative characterization of molecular order and mobility in ordered soft matter^31,32^. Due to their well-defined orientational and distance dependence, dipolar couplings have been proven to be sensitive probes of conformational changes and mobility of flexible molecules. We apply this technique to investigate properties of symmetric dicationic ionic liquid crystal, 3,3′-(1,6-hexanediyl)bis(1-dodecylimidazolium)dibromide (C_6_(C_12_im)_2_Br_2_) which exhibits a layered smectic phase in a wide temperature range from 63 to 132 °C.

## Results and discussion

### Director alignment and 1D NMR spectra

Anisotropic molecular interactions in liquid-crystalline state induce long-range molecular orientation along a common director. Bulk LC samples exposed to a magnetic field *B*_0_, often demonstrate a uniform or partial director alignment. The potential energy $P \propto - \Delta \chi B_{0}^{2} (3\cos^{2} \alpha - 1)$, which gives rise to an orientating torque, depends on the anisotropy ∆χ of the magnetic susceptibility tensor of material, and the angle α between the director and the magnetic field vector^33^. Macroscopic orientability is important property exploited in the development of functional mesogenic materials. In imidazolium-based long-chain ILs, exhibiting negative ∆χ, the interaction with the magnetic field is minimized when the director tends to line-up in a plane perpendicular to the magnetic field. The viscous torque opposes the director reorientation. While low-viscosity LCs (such as nematics) align spontaneously in NMR magnets, highly viscous smectic phases are more difficult to orient. It has been previously noted that single-chain monocationic ILCs can be aligned in a strong magnetic field of the order of 10 T upon cooling from the isotropic phase even at a relatively fast cooling rate of 25 °C/min^34^. However, DIL samples display more than an order of magnitude higher viscosity compared to that in mono-cationic analogues^19^. We found that in order to achieve a good alignment of the sample C_6_(C_12_im)_2_Br_2_ in the magnetic field of our NMR spectrometer, a very slow cooling rate of < 1 °C/min was essential in the vicinity of the phase transition temperature. Aligned samples can be studied by a high-resolution solid-state ^13^C NMR at static sample condition, that is, without magic angle sample spinning (MAS)^32^. This expands the range of suitable structural NMR methods and often provides a higher spectral resolution and sensitivity, as well as simplifies spectra analysis in terms of spin interactions.

In several reports on this and analogous materials, the layered mesophases were assigned as smectic A^6,8–10^. We note that in work Yang et al.^7^ the mesophase of C_6_(C_12_im)_2_Br_2_ was designated as smectic C phase, but it probably concerns smectic A phase as reported by Bara et al.^6^ The mesophase assignment as smectic C phase in this material was questioned in the review^1^. Furthermore, the reported enthalpy value of the isotropic-smectic transition of ~ 1 kJ/mol is representative of the smectic A phase of similar samples^7^. It is notable that the smectic C phase is rarely observed for ILCs^1^. In smectic A, the normal to the layer planes coincides with the director, while in smectic C it deviates with a uniform molecular tilt angle. This difference, however, does not affect the analysis and applied models of the orientational ordering in the studied sample, since, irrespective of the phase type, the director and the molecular axes align perpendicular to the magnetic field direction. The external field acts on the director and has no effect on the structure of the smectic layers^35,36^.

**Figure 1:**
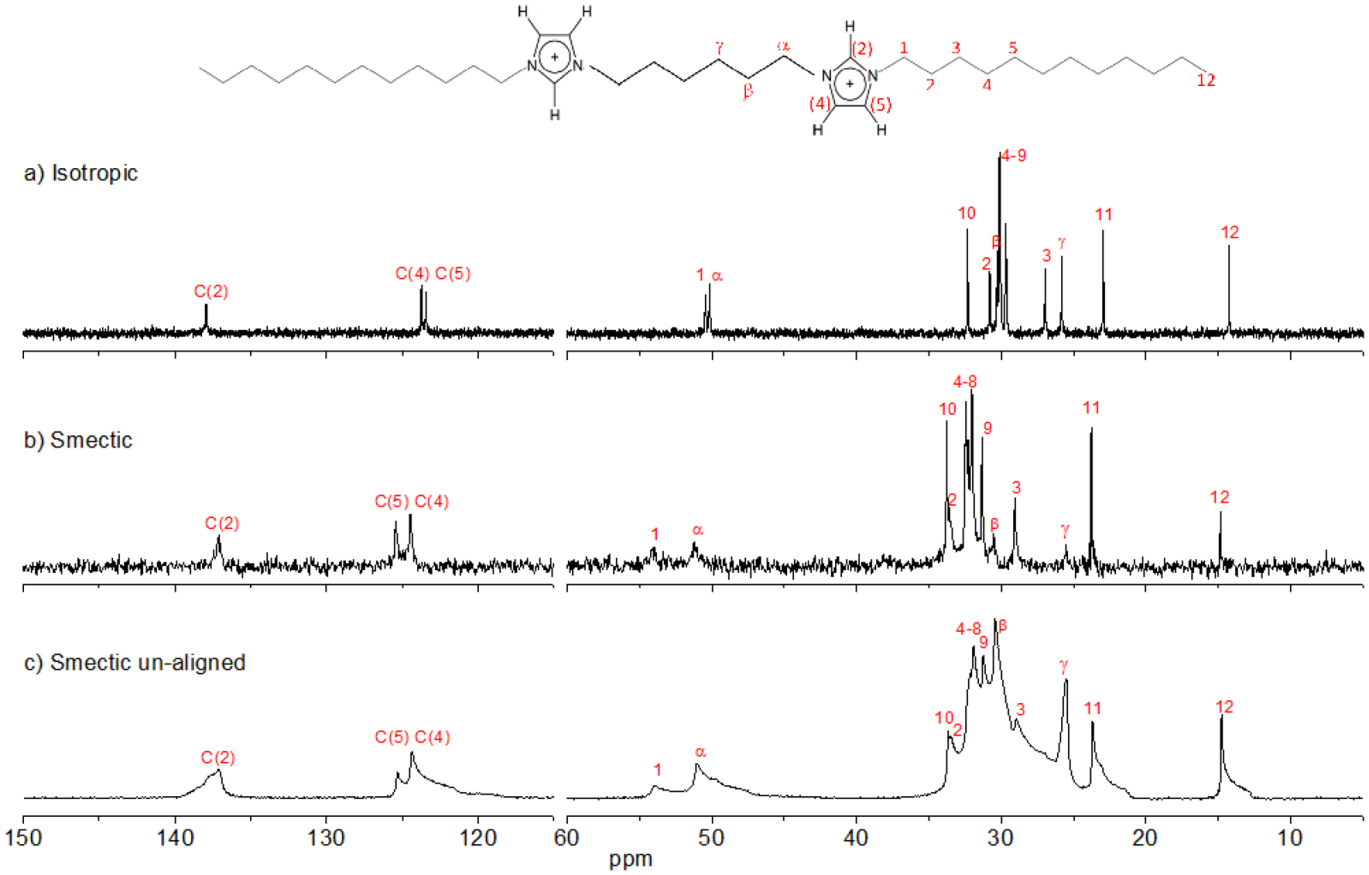
^13^C NMR spectra of C_6_(C_12_im)_2_Br_2_ in the isotropic phase at 140 °C (**a**) and smectic phase (**b**, **c**) at 111 °C. The spectrum (**b**) was recorded in the aligned mesophase using a single scan with cross-polarization (CP) enhancement. The spectrum (**c**) was recorded in the un-aligned mesophase with the number of scans 1024 and using nuclear Overhauser effect (NOE) enhancement. The cation structure with atom labeling is shown on the top.

The degree and direction of the sample alignment can be judged by inspection of the ^13^C NMR spectra in the isotropic (Fig. 1a) and smectic phases (Fig. 1b,c). The observed sharp ^13^C spectral lines in the aligned smectic phase indicated that a high degree of the director alignment was achieved at a low cooling rate 0.5 °C/min (Fig. 1b). Faster cooling rate led to less-resolved and lower-intensity spectral peaks (spectra not shown). The direction of the alignment was established from the comparison of the ^13^C spectra of the aligned sample and the sample prepared with a random director distribution (Fig. 1b,c, respectively). The random 3D director distribution was achieved by cooling the sample from the isotropic phase outside the NMR magnet. Due to the high viscosity of the smectic phase, the domains of different orientations do not reorient when the sample is placed in the magnetic field. The chemical shift tensor in liquid crystals is characterized by two principal components $\delta_{||}^{LC}$ and $\delta_{ \bot }^{LC}$ corresponding to chemical shifts for the LC domains with director along and orthogonal to the magnetic field, respectively^31,37^. The observed ^13^C chemical shift in the aligned DIL sample corresponded to the component $\delta_{ \bot }^{LC}$. The carbon signal positions in the isotropic phase in Fig. 1a matched the isotropic average $\delta_{i} = (\delta_{||}^{LC} + 2\delta_{ \bot }^{LC} )/3$ of respective chemical shift anisotropy (CSA) tensors.

The assignment of the carbon-13 peaks in the isotropic phase was obtained from 2D correlation spectra measured in solution and in the bulk isotropic state (Supplementary Figs. S1 and S2). The spectral assignment in the smectic phase was verified by the ^1^H–^13^C heteronuclear correlation spectra (HETCOR) in the aligned mesophase (see Supplementary Fig. S3a). Note the opposite order of the signals C(4) and C(5) observed in isotropic and smectic phases (Fig. 1).

### ^13^C–^1^H dipolar couplings and C–H bond order parameters

**Figure 2:**
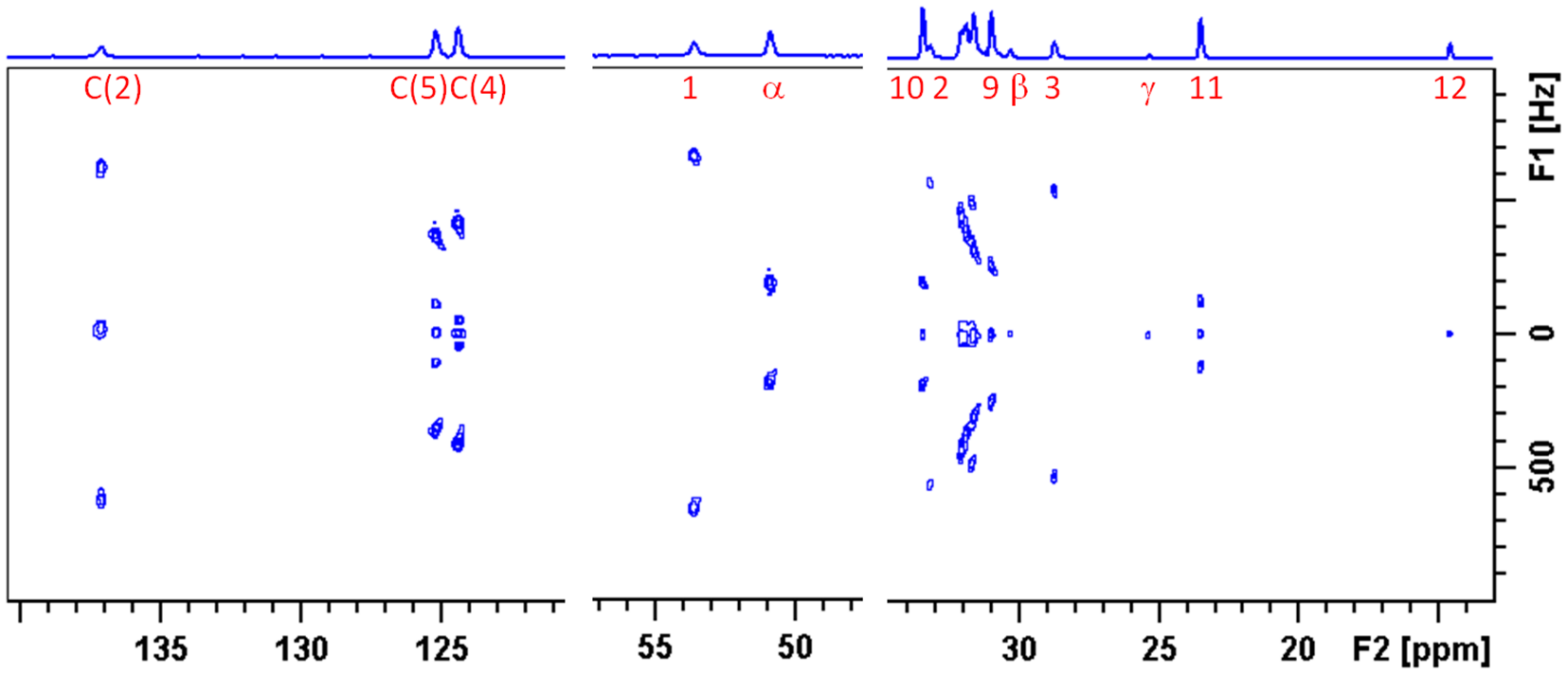
2D ^13^C–^1^H PDLF spectrum of C_6_(C_12_im)_2_Br_2_ sample in the smectic phase at 122 °C.

Contour plot of a representative 2D ^13^C–^1^H dipolar spectrum recorded by two-dimensional (2D) proton detected/encoded local field (PDLF) NMR spectroscopy^38^ is shown in Fig. 2. For each pair of mirror sites in the symmetric molecular parts, only one set of splittings was observed. For most of the carbon positions in the side-chains and imidazolium core, the spin couplings between directly bound ^13^C and ^1^H spins are well resolved. No splittings were observed for the carbons β and γ in the spacer and the terminal methyls. The aromatic carbons C(4) and C(5) exhibit, in addition to large splittings due to couplings with directly bound protons, also smaller splittings assigned to C(4)–H(5) and C(5)–H(4) couplings.

The spectral splitting $\Delta \nu$ in the dipolar dimension of the PDLF spectra is contributed by the pair-wise direct dipolar coupling $d_{CH}$ and indirect spin coupling $J_{CH}$:

1 $$ \Delta \nu = k(2d_{CH} + J_{CH} ) $$

(*k* ≈ 0.418 ± 0.002 is the heteronuclear dipolar scaling factor of the homonuclear decoupling sequence^39^ used in the present study). Only absolute values of the splittings $\Delta \nu$ are obtained from the symmetric doublets in the PDLF spectra. The magnitudes of the $J_{CH}$ couplings were measured in proton-coupled ^13^C spectra in the isotropic phase (the sign of a single-bond $J_{CH}$ coupling is positive^40^). The negative signs of the dipolar couplings of the aliphatic carbons were confirmed by comparison to the natural abundance deuterium (NAD) NMR spectrum (Fig. S4) using a previously developed approach^26,41^. The condition relating the quadrupolar splitting and dipolar coupling for aliphatic sites, $\Delta \nu_{Q} /d_{CH} \approx 11.7$^41^, could be satisfied only assuming negative signs of $d_{CH}$ in Eq. (1) (see details in Supplementary Material, Section S4). This relationship was also used to estimate dipolar coupling constants in the spacer. Comparison of obtained quadrupolar and dipolar couplings is shown in Supplementary Table S1. The signs of the dipolar couplings of the aromatic carbons were obtained by comparison to dipolar spectra recorded under MAS condition, using the procedure described previously^26^ (dipolar MAS spectra and details of the procedure are given in Supplementary Material Section S5).

The dipolar coupling constant $d_{CH} {\kern 1pt}$ is an orientation average of the rigid lattice coupling constant $b_{CH} = - (\mu_{0} /8\pi^{2} )(\gamma_{H} \gamma_{C} \hbar /r_{CH}^{3} )$ ($r_{CH}$ is atomic distance and $\gamma_{H} ,\,\;\gamma_{C}$ are gyromagnetic ratios). A simplified description of the averaging effect of anisotropic motion on spin interactions can be applied assuming an approximately cylindrical molecular symmetry and a large time scale separation of molecular reorientations and conformational dynamics^31,37^. The angular fluctuation of long molecular axes about the director is characterized by the molecular order parameter $S = \left\langle {(3\cos^{2} \theta_{MN} - 1)/2} \right\rangle$, where $\theta_{MN}$ is the instantaneous angle between the long molecular axis *M* and the director *N*). For a ^1^H–^13^C spin pair within a molecule, a local order parameter

2 $$ S_{CH} = \left\langle {(3\cos^{2} \theta_{PM} - 1)/2} \right\rangle S $$

is introduced, with the angle $\theta_{PM}$ defining the alignment of the internuclear vector (principal axes frame *P*) in the molecular frame *M*. The dipolar coupling in mesophase is given by

3 $$ d_{CH} = b_{CH} \,S_{CH} {\kern 1pt} (3\cos^{2} \theta_{NL} - 1)/2 $$

The angle $\theta_{NL}$ accounts for the tilt of the director *N* to the magnetic field ***B***_0_ (magnetic field direction defines the laboratory frame axis *L*). In our sample, aligned at the right angle to the magnetic field, the angular factor is $(3\cos^{2} \theta_{NL} - 1)/2 = - 0.5$. Thus, bond order parameters were calculated as

4 $$ S_{CH} = \,d_{CH} /( - 0.5b_{CH} ){\kern 1pt} $$

The accepted values for the dipolar coupling constants in the interaction frame, $b_{CH}$, with account for vibration effects, are − 21.5 kHz and − 22 kHz for aliphatic and aromatic sites, respectively^42,43^.

### Side-chain order

**Figure 3:**
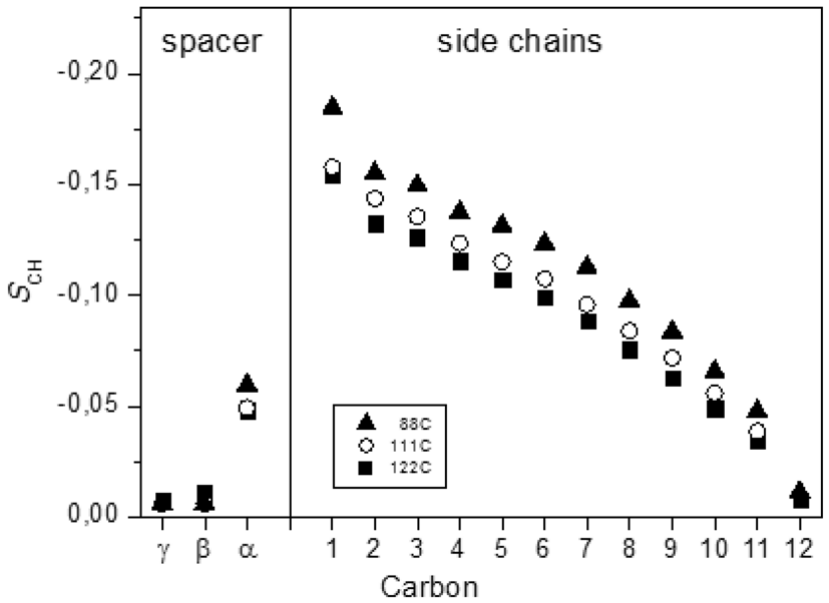
Local bond order parameters of the aliphatic carbons in the sample C_6_(C_12_im)_2_Br_2_ in the smectic phase.

Obtained bond order parameters of the C_6_(C_12_im)_2_Br_2_ sample at several temperatures in the smectic phase are displayed in Fig. 3. The order parameters decreased towards the terminal methyl groups as expected in the presence of chain conformational dynamics by trans/gauche isomerization. Qualitatively similar behavior was observed for monocationic single and double chain analogs^24–27^. The rest of the chain is more dynamic, however, it is forced to align on average with the director due to steric restrictions imposed by neighboring chains. Overall lower $S_{CH}$ values in the DILC compared to symmetric monocation analogue^27^ may indicate decreasing phase order parameter *S*.

The order parameter of the first carbon C1, having only limited conformational freedom, is primarily determined by the alignment of the imidazolium ring^26,27^. For example, a strongly reduced $S_{CH} ({\text{C}}1){\kern 1pt}$ value was found in the symmetric cation C_12_imC_12_ exhibiting ring plain nearly orthogonal to the cation long axis^27^. On the other hand, a high value $S_{CH} ({\text{C}}1){\kern 1pt}$ is indicative of an extended cation conformation^24–26^. More details on ring orientation are obtained from the analysis of the dipolar couplings in the aromatic core as described below.

### Spacer order

Carbons in the flexible spacer moiety exhibit very low C-H bond order parameters including that for the α group. Splittings for the methylenes β and γ were not resolved in the PDLF spectrum. Therefore corresponding order parameters were estimated from the splitting in the NAD spectrum (Section S4 in Supplementary Information). The low order in the spacer can be explained by intensive conformational dynamics of the spacer segments and/or by a specific alignment of the spacer. We performed density functional theory (DFT) analysis of dication structure for a number of conformations of the spacer. The range of considered geometries was limited by the requirement of large values of the order parameters $S_{CH}$ of the side chain segments in the vicinity of the imidazolium ring (according to experimental observations, Fig. 3) and also by taking into account the signs and relative magnitudes of the $S_{CH}$ values in the imidazolium moiety (Table 1). However, we were not able to find a particular single conformer that exhibited $S_{CH}$ values for spacer as low as found in the experiment. Thus, the dynamic origin of low $S_{CH}$ values in the spacer has to be assumed. For example, with three-fold reorientation around N–Cα bond, the order parameter $S_{CH}$ =  − 0.09 is obtained for the α group. For β and γ carbons, a further decrease of the $S_{CH}$ magnitude is expected due to trans/gauche conformational dynamics of the spacer segments. We also note that the spacer carbons exhibit a rather small value of CSA when compared to that of the side chains (Fig. 1c). Since the main principal axes of CSA and dipolar interactions in the methylene group are nearly orthogonal^44^, the corresponding angular parameters of two interaction tensors in the rigid spacer are unlikely to be simultaneously small. Hence, observed small order parameters are resulted from conformational dynamics and not from a particular alignment of the spacer segments. Since the flexible spacer is confined in a space between bulky charged groups, its conformational freedom stems from an increased free volume in the ionic sublayer space as compared to the monocationic counterparts. In contrast, the conformational dynamics of the side chains is limited due to steric constraints imposed by interdigitated neighboring chains^7^.

**Table 1:** Experimental C–H local order parameters $S_{CH}$ in imidazolium ring and cation order parameter *S*.

*T* (°C)	$S_{C(2)H(2)}$	$S_{C(4)H(4)}$	$S_{C(5)H(5)}$	$\left| {S_{C(4)H(5)} } \right|$	$\left| {S_{C(5)H(4)} } \right|$	*S*
122	− 0.146	0.081	− 0.089	0.081	0.186	0.324
111	− 0.156	0.088	− 0.093	–	0.191	0.353
88	− 0.167	0.106	− 0.104	–	0.230	0.404

Signs of the parameters $S_{C(4)H(5)}$ and $S_{C(5)H(4)}$ were not available from the experimental data. The model analysis suggested positive signs for these two parameters.

### Alignment of the imidazolium rings

**Figure 4:**
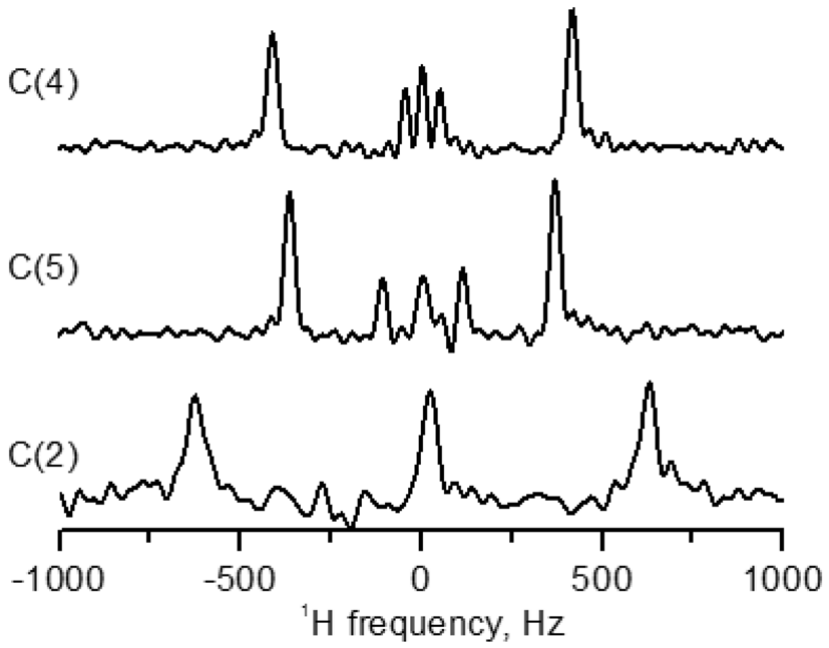
Dipolar cross sections for the carbons in imidazolium ring obtained from 2D PDLF spectrum of C_6_(C_12_im)_2_Br_2_ in smectic phase at 122 °C.

The dipolar cross-sections for the imidazolium ring carbons for the sample C_6_(C_12_im)_2_Br_2_ in the smectic phase at 122 °C are displayed in Fig. 4. Doublets with larger splittings are due to couplings with directly bound protons. Additional smaller splittings for the carbons C(4) and C(5) correspond to longer-range C-H couplings in the spin pairs C(4)–H(5) and C(5)–H(4). Assignment of these remote couplings was obtained in the heteronuclear correlation ^13^C–^1^H spectrum with dipolar evolution (HETCOR-PDLF) shown in Supplementary Fig. S3b. Estimated values of the local order parameters of C–H pairs, using Eq. (2), are listed in Table 1.

The relative values of the local bond order parameters in the imidazolium core of the DILC are comparable to those found in the analogous ILCs with single-chain monocation C_12_mim^26^. The alignment of the imidazolium ring in the C_12_mim cation corresponded to the extended DFT-optimized conformation with the ring plane as near as possible parallel to the molecular axis. Other low-energy DFT-optimized structures have also been reported, with the ring plane perpendicular to the chain axis (as observed, e.g., in the symmetric double-chain monocationic sample)^27^ and with the ring plane parallel to the chain backbone plane^26^. These other geometries, however, are not consistent with the experimental magnitudes and signs of the local order parameters in Table 1, as well as with a relatively high $S_{CH}$ value for the first methylene group of the side chains (Fig. 3).

Detailed information on the ring orientation in DIL mesophase was obtained from the numerical fitting procedure. The ring plane alignment in the molecular frame was varied to simultaneously fit the experimental values of the five order parameters in Table 1 to the equation

5 $$ S_{CH}^{(i)} = 0.5\left\langle {3\cos^{2} \theta_{PM}^{(i)} - 1} \right\rangle S, $$

where $\theta_{PM} (\alpha ,\beta ,\gamma )$ defines the angle between C–H vector (principal frame *P*) and long molecular axis M, and $(\alpha ,\beta ,\gamma )$ present Euler angles. The ring geometry was taken from the DFT-optimized structure of dication. To reduce the number of fitting parameters in the model, the biaxiality term $(S_{XX} - S_{YY} )$ of the ordering matrix was neglected, tentatively assuming $S_{ZZ} \gg \left( {S_{XX} - S_{YY} } \right)$^41^. We have previously shown that such an approximation was adequate to correctly predict signs and relative magnitudes of the bond-order parameters in other imidazolium-based cations^26,27^.

**Figure 5:**
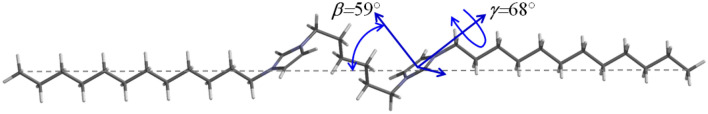
Schematic illustration of the imidazolium ring alignment in the DIL. This DFT-optimized structure is consistent with the experimental order parameters found in the imidazolium ring and for C1 methylenes. Other possible lower energy structures with the ring planes perpendicular to the all-trans side-chain axis or parallel to the side-chain backbone plane^26,27^ are not consistent with the experimental data.

The schematic molecular structure illustrating the ring orientation is shown in Fig. 5. Indicated best-fit values of the tilt angles of the ring axes correspond to experimental data at 111 °C. For simplicity, the long cation axis is shown as the line connecting terminal methyl carbons in all-trans side chains. Knowing the explicit position of the molecular axis, which is conformation-dependent, is not required for the analysis since the experimental local order parameters are given in the director reference frame. The effect of the angular fluctuations of the molecular axis with respect to director is included in the order parameter in Eq. (5). Considering the structural moiety which includes one imidazolium ring and adjacent side-chain, the obtained best-fit ring alignment indeed resembles the extended structure of the C_12_mim monocation^26^. For example, the analogous fit of the data in C_12_mimBr at 79 °C from work^25^ resulted in the values *β* = 52° and *γ* = 73°, not much different from those in the DIL sample.

### Order parameter

Estimated *S* values in this and previously studied ILCs were based on certain assumptions on the model structures of the cations in mesophase^24–27^. In each case, we assumed that (i) the cation adopts on average an elongated structure, (ii) for the entire cation there exists one unique preferred direction of orientation, and (iii) the alignment of the imidazolium ring is consistent with one of the DFT-optimized low-energy structures. In all studied materials, we obtained a good consistency of a large set of experimental and model bond order parameters *S*_CH_, which were used to estimate the molecular order parameter *S*. In the DILC sample, the calculated order parameter *S* varies in the range 0.32…0.40 depending on temperature (Table 1). These values, which are significantly lower than the typical order parameter values in classical smectic phases of neutral molecules^45,46^, can be compared to monocationic ionic liquid crystals with single and double chains. The large molecular size and higher anisotropy of the cation shape in the DIL sample should generally contribute to increasing molecular order. Indeed, the order parameter is higher when compared to that in the smectic C_12_mimBr based on a single-chain monocation^25^. On the other hand, the order parameter in the DIL is significantly lower when compared to *S* ~ 0.54…0.61 obtained in its monocationic double-chain counterpart C_12_imC_12_Br having lower molecular weight^27^. This behavior can be understood by considering the interplay between major intermolecular forces contributing to mesophase stabilization.

It may be recalled that a high orientational order *S* > 0.6 is essential for the stability of mesophases formed by *neutral* molecules because smectic alignment is primarily driven by weak van der Waals interactions^45^. In contrast, smectic layers in ILCs can form with a low orientational order because of the presence of strong electrostatic and hydrogen bonding interactions in the ionic sublayer. Induced segregation of polar and apolar domains contributes to the phase stabilization of less ordered molecules. Smectic phases with *S* value as low as 0.2 have been found in single-chain imidazolium-based monocationic salts^24–26^, as has been also confirmed by molecular dynamics simulation^47^. We have recently shown that a modification of the cation structure by adding a second symmetrically substituted long chain altered the equilibrium between the interactions in polar and hydrophobic sublayers^27^. Due to the enhanced contribution of van der Waals forces in the double chain cations, the order parameter has increased dramatically. However, the interfering effect of larger cation size and anisotropy of the double chain ILCs was difficult to estimate and disentangle from the effect of shifted balance between interaction forces. Present results provided strong support for the interaction model based on a delicate equilibrium of inter-particle forces driving smectic ordering. Double-charge cation coordinates a larger number of anions which also contribute with more possibilities to form hydrogen bonds in the dicationic moiety. Importantly, H-bonds can be formed also with weakly acidic hydrogens in the aliphatic chains^22^. With increased interaction energy in the polar domain and induced segregation of polar and apolar moieties, the layered structure is stabilized despite an overall higher disorder in cation orientation and conformation. Notably, bond order parameters in the side chains in DIL smectic phase are consistently lower compared to those in double-chain monocation, also suggesting a diminishing role of van der Waals forces. This can be due to larger excluded volume induced by changed chain interdigitation in bilayers^7^. A strong difference in size and shape of cation and anion in ILCs contribute to excluded volume effect on mesophase stability. Also, the density of the ionic liquids decreased with increasing linkage chain length^5^.

## Conclusions

We have studied molecular conformation and orientational order of dicationic ionic liquid C_6_(C_12_im)_2_Br_2_ exhibiting thermotropic smectic mesophase. The material belongs to a new class of gemini ionic liquid crystals possessing promising physicochemical properties. Despite high viscosity and relatively large molecular weight, the studied DIL in the smectic phase can be macroscopically aligned in the magnetic field of the order of 10 T when cooled slowly from the isotropic liquid state. Thus the sample was suitable for studies by high-resolution solid-state NMR at static sample conditions. Advanced dipolar NMR spectroscopic techniques were applied to obtain the conformational structure and orientational order of the dication. We have estimated the local bond order parameters and alignments of linker and side-chains and imidazolium ring in mesophase. We found that the side-chain ordering and the alignment of imidazolium rings are similar to those in analogous single-chain monocationic salts. To explain this finding, we hypothesized that the presence of a highly dynamic spacer effectively “decouples” the motion of two imidazolium rings. Indeed, the spacer exhibited liquid-like conformational dynamics with vanishing residual ordering of C–H bonds.

Overall dication reorientation in the smectic phase was characterized by rather low values of the orientational order parameter *S*. This result emphasized the trend in the orientational order of cations and provided us with an important piece of knowledge to present a consistent picture of interparticle interactions driving and stabilizing smectic assembly in ILCs. Our approach to separate the contribution of different forces was based on a concept of controllable tuning of a specific force and monitoring induced structural and orientational order changes at the molecular level. This strategy has provided us with physical insights into the complex interplay of different intermolecular forces driving mesophase formation. The possibility to control specific contributions in the complex energy landscape of intermolecular interactions in ILCs is important for the rational and efficient design of ILC-based functional materials.

Further insights into ion dynamic properties can be obtained from studies of translational diffusion in DILCs. The translational dynamics of ions is related to conductivity and as such is of high interest in the context of electrochemical applications. In mesophases, the self-diffusion and ion transport are strongly anisotropic which opens the possibility to design low-dimensional electrolytes^48,49^. High translational anisotropy is also indicative of phase stability. Director alignment in the magnetic field makes it possible to access the diffusion anisotropy using pulsed-field-gradient NMR techniques^50^. Diffusion studies of ILCs are currently in progress in our laboratory.

## Methods

Synthesis of symmetrical dicationic ionic liquid crystal C_6_(C_12_im)_2_Br_2_ was carried out following the reported literature procedures^5–7,19^. One mmol of 1,6 di-bromohexane was added dropwise to a 1-dodecylimidazole 2.2 mmol acetonitrile solution. The reaction mixture was stirred under a nitrogen atmosphere in a reflux condenser for 3 days. The solution was subsequently condensed by evaporation and the product was collected and washed several times with diethyl ether. The obtained white precipitate was purified by recrystallization in an ethanol/diethyl ether mixture. The purified material was dried under a vacuum. The compound was characterized by proton and carbon solution NMR.

Bulk C_6_(C_12_im)_2_Br_2_ sample exhibited stable smectic phase in a wide temperature range with the phase transition temperatures $T_{Cr \to Sm} = 63\;^\circ {\text{C}}$ (heating run at 5 °C/min) and $T_{Iso \to Sm} = 132\;^\circ {\text{C}}$ (cooling run at 0.5 °C/min). Transitional temperatures were determined by observing ^1^H NMR spectra during heating or cooling. Somewhat narrow mesophase range as compared to that reported in other studies^6,7^ was attributed to lower water content in our sample. It has been shown that water presence leads to a wider temperature range of mesophase stabilization^51–53^. The ^1^H spectrum in the isotropic phase indicated the presence of 5 mol% water in the sample (Supplementary Fig. S6). This small amount of water was expected to have a negligible effect on the orientational order of ions^1,51,53^.

NMR experiments were performed using a Bruker 500 Avance III spectrometer at Larmor frequencies of 500.1, 125.7, and 76.8, for ^1^H, ^13^C, and ^2^H, respectively. NMR spectra were recorded using a custom-modified solution state multinuclear 5 mm probe-head. The ^1^H, ^13^C, and ^2^H 90° pulse lengths were 8, 13, and 6 μs, respectively. For heteronuclear proton decoupling in the mesophase, Spinal64 sequence^54^ was used with the proton nutation frequency of 25 kHz. To enhance the intensity of the ^13^C signal, proton-to-carbon cross-polarization (CP) was applied with nutation frequencies of about 20 kHz and contact time optimized in the range of 3–12 ms. Dipolar ^13^C–^1^H spectra were recorded using two-dimensional (2D) proton detected/encoded local field (PDLF) NMR spectroscopy^38^. Proton homonuclear decoupling was achieved by BLEW-48 multiple-pulse sequence^39^ with a nutation frequency of 31.2 kHz. The evolution time in the indirect time domain was incremented with 192 μs in 256 steps, at each with 4 collected transients. Natural abundance deuterium (NAD) NMR spectra were recorded by quadrupolar echo sequence with echo delay of 30 μs and in the presence of broadband proton decoupling. The sample temperature was regulated with an accuracy of 0.1 °C. Decoupling powers, irradiation times, and repetition delays were adjusted to limit sample heating effects to < 0.5 °C.

Density functional theory computational analysis was performed using the Spartan’18 program^55^. Geometry optimization was carried out for isolated dication (*in vacuo*) with B3LYP/6-311++G** theory level. Several structures were examined with different alignments of the imidazolium rings and conformations of the flexible spacer.

## Supplementary Information

Supplementary Information

## References

[1] Goossens K, Lava K, Bielawski CW, Binnemans K (2016). Ionic Liquid crystals: versatile materials. Chem. Rev..

[2] Axenov KV, Laschat S (2011). Thermotropic ionic liquid crystals. Materials.

[3] Fernandez AA, Kouwer PHJ (2016). Key developments in ionic liquid crystals. Int. J. Mol. Sci..

[4] Douce L, Suisse JM, Guillon D, Taubert A (2011). Imidazolium-based liquid crystals: a modular platform for versatile new materials with finely tuneable properties and behaviour. Liq. Cryst..

[5] Anderson JL, Ding RF, Ellern A, Armstrong DW (2005). Structure and properties of high stability geminal dicationic ionic liquids. J. Am. Chem. Soc..

[6] Bara JE (2010). Thermotropic liquid crystal behaviour of gemini imidazolium-based ionic amphiphiles. Liq. Cryst..

[7] Yang M, Stappert K, Mudring AV (2014). Bis-cationic ionic liquid crystals. J. Mater. Chem. C.

[8] Ilinca TA, Pasuk I, Circu V (2017). Bis-imidazolium salts with alkyl sulfates as counterions: Synthesis and liquid crystalline properties. New J. Chem..

[9] Maximean DM, Circu V, Ganea CP (2018). Dielectric properties of a bisimidazolium salt with dodecyl sulfate anion doped with carbon nanotubes. Beilstein J. Nanotech..

[10] Pana A (2014). Liquid crystals based on silver carbene complexes derived from dimeric bis(imidazolium) bromide salts. RSC Adv..

[11] Causin V, Saielli G (2009). Effect of a structural modification of the bipyridinium core on the phase behaviour of viologen-based bistriflimide salts. J. Mol. Liq..

[12] Maton C, De Vos N, Stevens CV (2013). Ionic liquid thermal stabilities: decomposition mechanisms and analysis tools. Chem. Soc. Rev..

[13] Pietralik Z, Kolodziejska Z, Weiss M, Kozak M (2015). Gemini surfactants based on bis-imidazolium alkoxy derivatives as effective agents for delivery of nucleic acids: A structural and spectroscopic study. PLoS ONE.

[14] Zhou T (2013). High transfection efficiency of homogeneous DNA nanoparticles induced by imidazolium gemini surfactant as nonviral vector. J. Phys. Chem. C.

[15] Bhadani A (2016). Structural diversity, physicochemical properties and application of imidazolium surfactants: Recent advances. Adv. Colloid Interface Sci..

[16] Tabushi I, Yamamura K, Kominami K (1986). Electric stimulus-response behavior of liquid-crystalline viologen. J. Am. Chem. Soc..

[17] Beneduci A, Cospito S, Imbardelli D, De Simone BC, Chidichimo G (2015). n-Type columnar liquid crystal combining ionic and electronic functions. Mol. Cryst. Liq. Cryst..

[18] Aviles MD, Sanchez C, Pamies R, Sanes J, Bermudez MD (2019). Ionic liquid crystals in tribology. Lubricants.

[19] Shirota H, Mandai T, Fukazawa H, Kato T (2011). Comparison between dicationic and monocationic ionic liquids: Liquid density, thermal properties, surface tension, and shear viscosity. J. Chem. Eng. Data.

[20] Majhi D, Seth S, Sarkar M (2018). Differences in the behavior of dicationic and monocationic ionic liquids as revealed by time resolved-fluorescence, NMR and fluorescence correlation spectroscopy. Phys. Chem. Chem. Phys..

[21] Dong K, Zhang SJ (2012). Hydrogen bonds: A structural insight into ionic liquids. Chem. Eur. J..

[22] Hunt PA, Ashworth CR, Matthews RP (2015). Hydrogen bonding in ionic liquids. Chem. Soc. Rev..

[23] Welton T (2018). Ionic liquids: A brief history. Biophys. Rev..

[24] Dai J, Kharkov BB, Dvinskikh SV (2019). Molecular and segmental orientational order in a smectic mesophase of a thermotropic ionic liquid crystal. Crystals.

[25] Dai J, Majhi D, Kharkov BB, Dvinskikh SV (2019). NMR spectroscopic study of orientational order in imidazolium-based ionic liquid crystals. Crystals.

[26] Majhi D, Dai J, Komolkin AV, Dvinskikh SV (2020). Understanding ionic mesophase stabilization by hydration: a solid-state NMR study. Phys. Chem. Chem. Phys..

[27] Majhi D, Komolkin AV, Dvinskikh SV (2020). NMR spectroscopic studies of cation dynamics in symmetrically-substituted imidazolium-based ionic liquid crystals. Int. J. Mol. Sci..

[28] Wuckert E (2015). Photoresponsive ionic liquid crystals based on azobenzene guanidinium salts. Phys. Chem. Chem. Phys..

[29] Quevillon MJ, Whitmer JK (2018). Charge transport and phase behavior of imidazolium-based ionic liquid crystals from fully atomistic simulations. Materials.

[30] Saielli G, Wang YT (2016). Role of the electrostatic interactions in the stabilization of ionic liquid crystals: Insights from coarse-grained MD simulations of an imidazolium model. J. Phys. Chem. B.

[31] Dong RY (2010). Nuclear Magnetic Resonance Spectroscopy of Liquid Crystals.

[32] Dvinskikh, S. V. in *Modern Methods in Solid-State NMR: A Practitioners’ Guide* (ed P. Hodgkinson) (Royal Society of Chemistry, 2018).

[33] de Gennes PG, Prost J (1993). The Physics of Liquid Crystals.

[34] Judeinstein P, Huet S, Lesot P (2013). Multiscale NMR investigation of mesogenic ionic-liquid electrolytes with strong anisotropic orientational and diffusional behaviour. RSC Adv..

[35] Meiboom S, Luz Z (1973). Nuclear magnetic resonance study of smectic phases. Mol. Cryst. Liq. Cryst..

[36] Wise RA, Smith DH, Doane JW (1973). Nuclear magnetic resonance in the smectic C phase. Phys. Rev. A.

[37] Dvinskikh SV, Sandström D, Zimmermann H, Maliniak A (2006). Carbon-13 NMR spectroscopy applied to columnar liquid crystals. Progr. Nucl. Magn. Reson. Spectrosc..

[38] Dvinskikh SV, Zimmermann H, Maliniak A, Sandström D (2003). Separated local field spectroscopy of columnar and nematic liquid crystals. J. Magn. Reson..

[39] Burum DP, Linder M, Ernst RR (1981). Low-power multipulse line narrowing in solid-state NMR. J. Magn. Reson..

[40] Breitmaier E, Voelter W (1990). Carbon-13 NMR Spectroscopy. High-Resolution Methods and Applications in Organic Chemistry and Biochemistry.

[41] Emsley JW (2008). A comparison of proton-detected C-13 local field experiments with deuterium NMR at natural abundance for studying liquid crystals. Liq. Cryst..

[42] Dvinskikh SV, Zimmermann H, Maliniak A, Sandström D (2004). Measurements of motionally averaged heteronuclear dipolar couplings in MAS NMR using R-type recoupling. J. Magn. Reson..

[43] Dvinskikh SV, Sandström D (2005). Frequency offset refocused PISEMA-type sequences. J. Magn. Reson..

[44] Schmidt-Rohr K, Spiess HW (1994). Multidimensional Solid-State NMR and Polymers.

[45] McMillan WL (1971). Simple molecular model for the smectic A phase of liquid crystal. Phys. Rev. A.

[46] Fung BM (1986). Nematic and smectic ordering of 4-n-octyl-4'-cyanobiphenyl studied by carbon-13 NMR. Mol. Cryst. Liq. Cryst..

[47] Saielli G (2012). MD simulation of the mesomorphic behaviour of 1-hexadecyl-3-methylimidazolium nitrate: Assessment of the performance of a coarse-grained force field. Soft Matter.

[48] Frise AE, Dvinskikh SV, Ohno H, Kato T, Furo I (2010). Ion channels and anisotropic ion mobility in a liquid-crystalline columnar phase as observed by multinuclear NMR diffusometry. J. Phys. Chem. B.

[49] Frise AE (2010). Ion conductive behaviour in a confined nanostructure: NMR observation of self-diffusion in a liquid-crystalline bicontinuous cubic phase. Chem. Commun..

[50] Dvinskikh SV (2020). Nuclear magnetic resonance studies of translational diffusion in thermotropic ionic liquid crystals. Liq. Cryst..

[51] Getsis A, Mudring AV (2008). Imidazolium based ionic liquid crystals: Structure, photophysical and thermal behaviour of [C(n)mim]Br·xH_2_O (n=12, 14; x=0, 1). Cryst. Res. Technol..

[52] Downard A (2004). Structural studies of crystalline 1-alkyl-3-methylimidazolium chloride salts. Chem. Mater..

[53] Puntus LN, Schenk KJ, Bunzli JCG (2005). Intense near-infrared luminescence of a mesomorphic ionic liquid doped with lanthanide beta-diketonate ternary complexes. Eur. J. Inorg. Chem..

[54] Fung BM, Khitrin AK, Ermolaev K (2000). An improved broadband decoupling sequence for liquid crystals and solids. J. Magn. Reson..

[55] Shao Y (2006). Advances in methods and algorithms in a modern quantum chemistry program package. Phys. Chem. Chem. Phys..

