# Supplementary material for: Ion conformation and orientational order in a dicationic ionic liquid crystal studied by solid-state nuclear magnetic resonance spectroscopy
Source: Sci Rep. 2021 Mar 16;11:5985. doi: 10.1038/s41598-021-85021-y (PMC7971035; doi:10.1038/s41598-021-85021-y)
Supplement: Supplementary file 1 — Supplementary Information [file 41598_2021_85021_MOESM1_ESM.pdf]

Supplementary Material

Ion Conformation and Orientational Order in a Dicationic Ionic Liquid Crystal Studied by Solid-State Nuclear Magnetic Resonance Spectroscopy

Debashis Majhi and Sergey V. Dvinskikh

S1. 2D spectra in solution

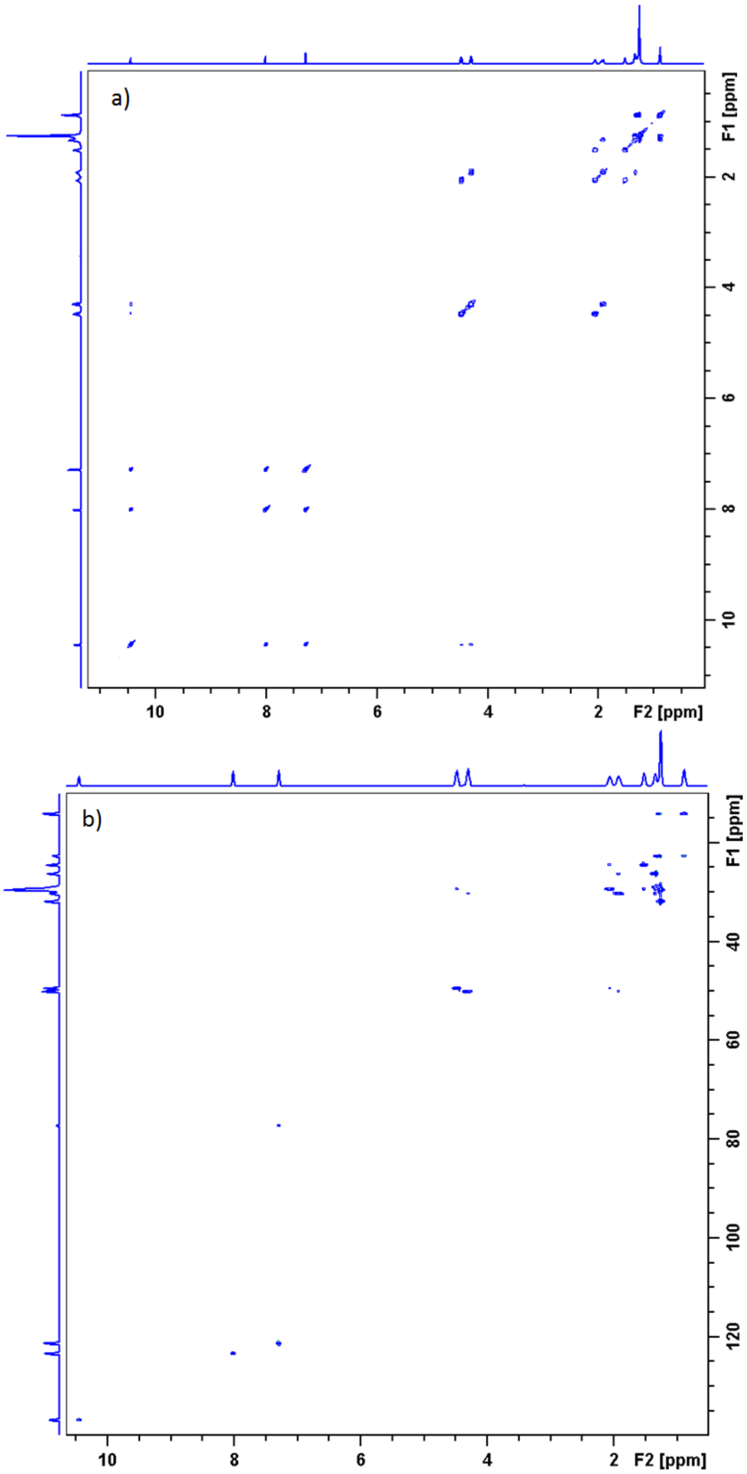

Figure S1. COSY (a) and HSQC (b) spectra of $C_6(C_{12}im)_2Br_2$ in chloroform solution.

S2. 2D spectra in the isotropic phase

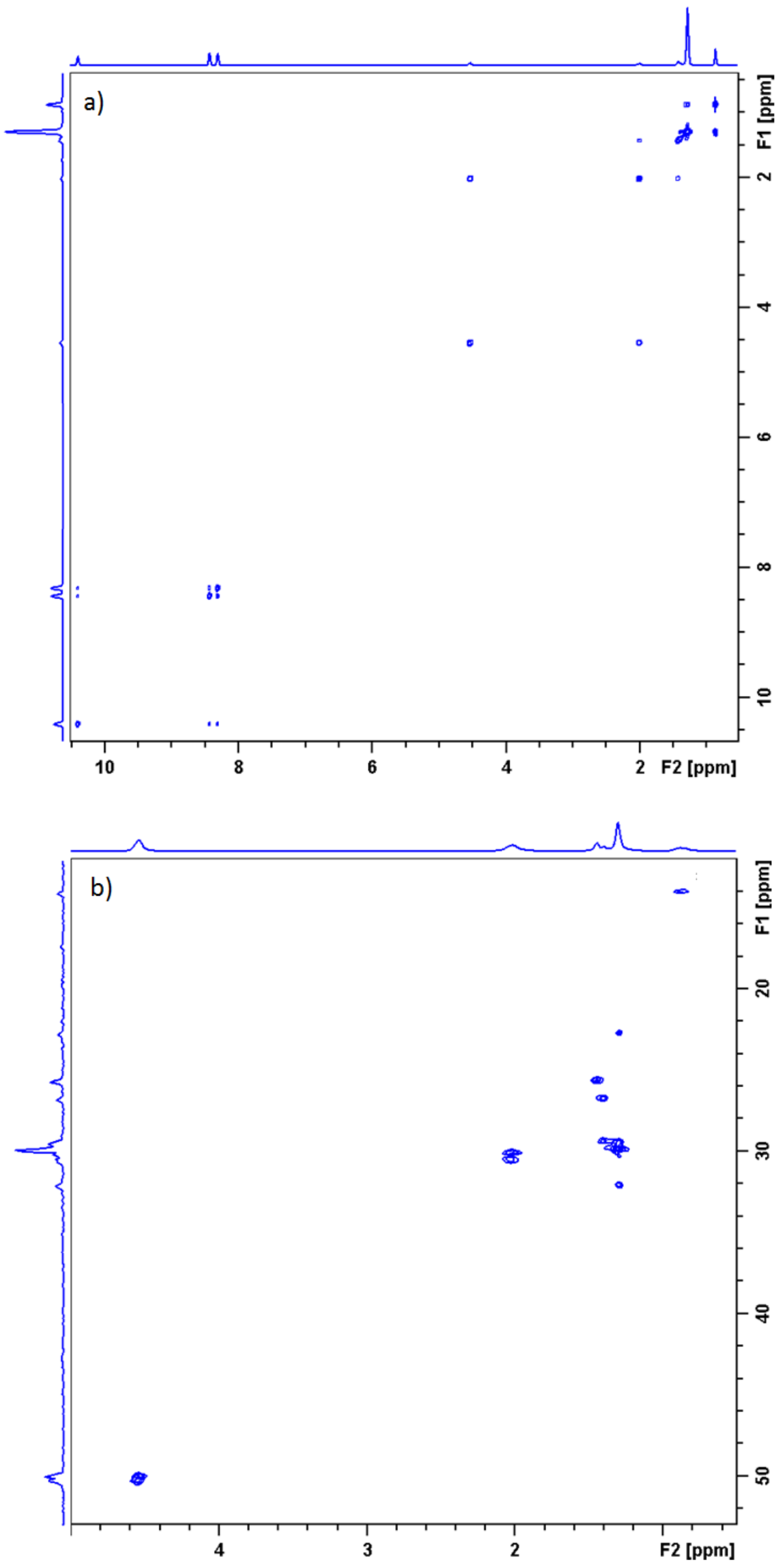

Figure S2. COSY (a) and HMQC (b) spectra of $C_6(C_{12}im)_2Br_2$ sample in bulk isotropic phase at 150 °C. In the HMQC spectrum, only the aliphatic region is shown.

S3. HETCOR spectra in the smectic phase

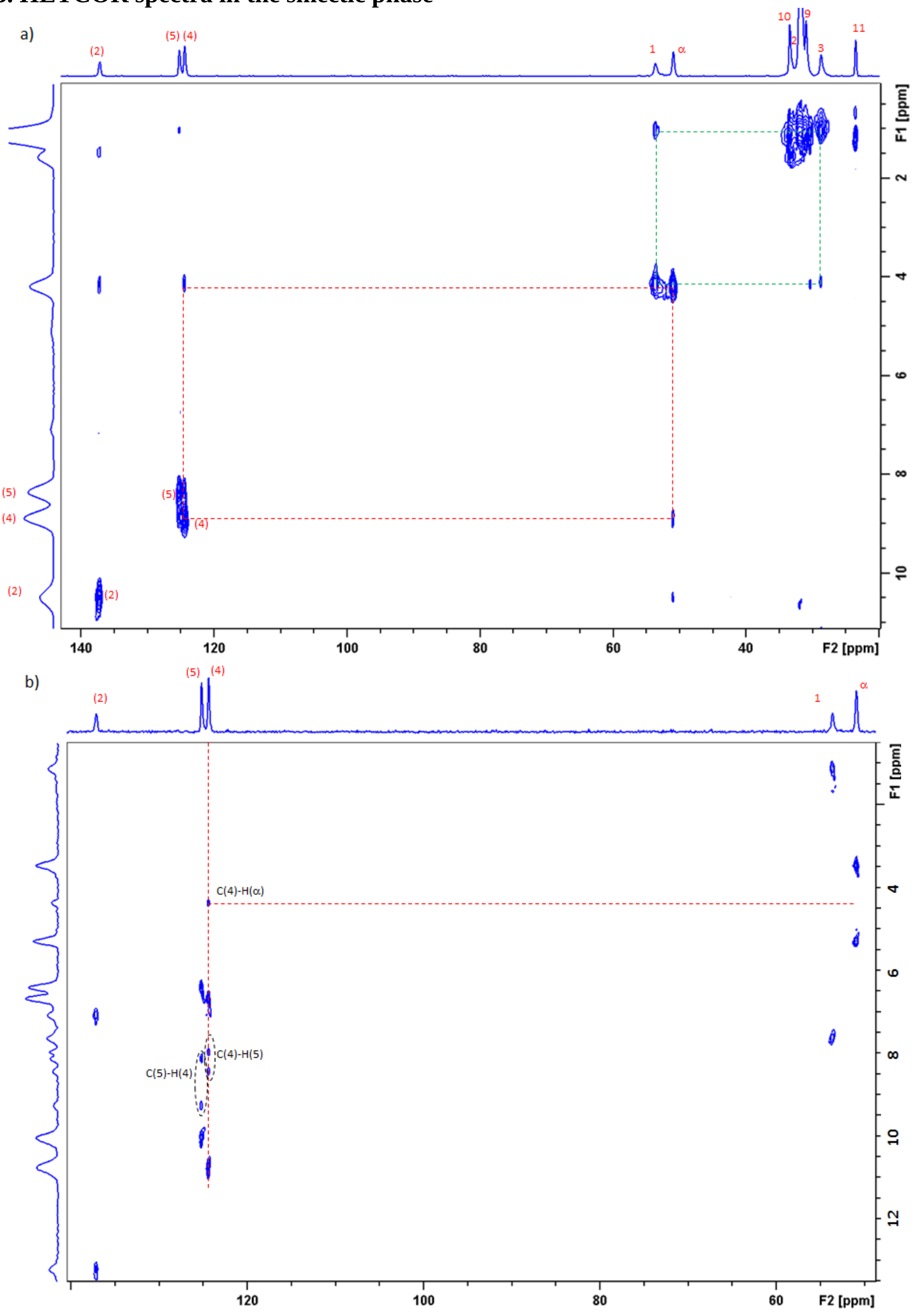

Figure S3. HECTOR (a) and HETCOR-PDLF (b) spectra of $C_6(C_{12}im)_2Br_2$ sample in the aligned smectic phase at 122 °C.

S4. Natural abundance deuterium NMR

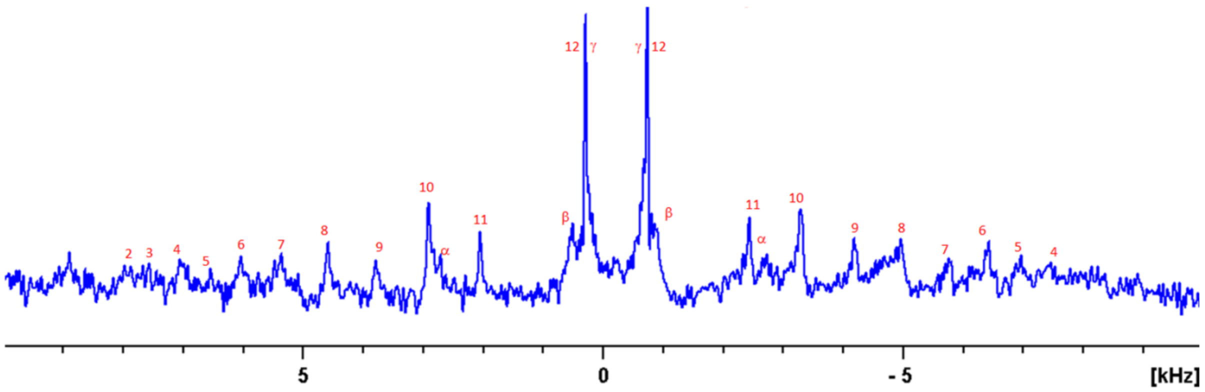

Figure S4. NAD NMR spectrum of $C_6(C_{12}im)_2Br_2$ sample in smectic phase at 122 °C. The assignment of the side chain methylenes in this spectrum is based on the comparison to the dipolar splittings in Fig. 2 (in the main text) which exhibit a monotonous decrease along the side chains. Signals from the imidazolium rings and first methylene in the side chains are not observed due to broader lines with lower signal-to-noise ratios and overlap with other lines.

Table S1. Comparison of the dipolar couplings calculated from the splittings in the PDLF experiment with those estimated from the quadrupolar splittings in the NAD experiment for the $C_6(C_{12}im)_2$ cation in the smectic phase at 122 °C.

Carbon	$\lvert d_{CH}^{(Q)} \rvert$,[a] Hz	$d_{CH}^{(PDLF)}$,[b] Hz
C1	-	-1656
C2	1393	-1424
C3	1337	-1357
C4	1239	-1242
C5	1156	-1156
C6	1065	-1065
C7	953	-951
C8	815	-814
C9	681	-679
C10	529	-527
C11	380	-370
C12[c]	87	-
Cα	479	-519
Cβ[c]	119	-
Cγ[c]	~80	-

[a] Estimated from quadrupolar splittings, assuming $\left|\Delta\nu_Q / d_{CH}\right| \approx 11.7$ [1].

[b] Calculated from equation S1, assuming negative sign of the dipolar coupling.

[c] The splitting was unresolved in the PDLF spectrum.

The procedure to infer the signs and magnitudes of the dipolar couplings of the aliphatic sites is based on the comparison of the dipolar coupling constants d_{CH}, estimated from the equation

$$\Delta\nu = k(2d_{CH} + J_{CH}) \qquad \text{(S1)}$$

to the corresponding quadrupolar splittings $\Delta\nu_Q$ measured in NAD spectrum. In a sample with the director perpendicular to the external magnetic field, the experimental dipolar coupling d_{CH} is related to the local bond order parameter S_{CH} as

$$d_{CH} = -(1/2)b_{CH} S_{CH} , \qquad \text{(S2)}$$

where b_{CH} is a rigid lattice dipolar coupling constant. Corresponding quadrupolar splittings for the deuterated site is given by

$$\Delta\nu_Q = -(3/4)\chi_Q S_{CH} , \qquad \text{(S3)}$$

where χ_Q is the quadrupolar coupling constant. With accepted experimental values b_{CH} =–21.5 kHz and χ_Q = 168 kHz for methylene sites[1-3], one obtains $|\Delta\nu_Q / d_{CH}| \approx 11.7$.[1] For the methylene carbons in the $C_6(C_{12}im)_2Br_2$ cation, this condition could be satisfied only by assuming the negative signs of the constants d_{CH} in Eq. (S1).

S5. Dipolar-recoupled magic-angle-spinning spectra

The dipolar spectra of the C-H pairs in the imidazolium moiety were recorded under the magic angle spinning (MAS) condition using the amplitude- and phase-modulated cross-polarization (APM-CP) dipolar recoupling scheme[4]. The splittings in the APM-CP spectra are given by $\Delta\nu = d_{CH} / \sqrt{2}$, where $\left(1/\sqrt{2}\right)$ is the scaling factor of the APM-CP sequence. Thus, comparing the spectral splittings obtained by the PDLF and APM-CP techniques (Table S2), the magnitudes and signs of the constants d_{CH} for the imidazolium C–H pairs were determined.

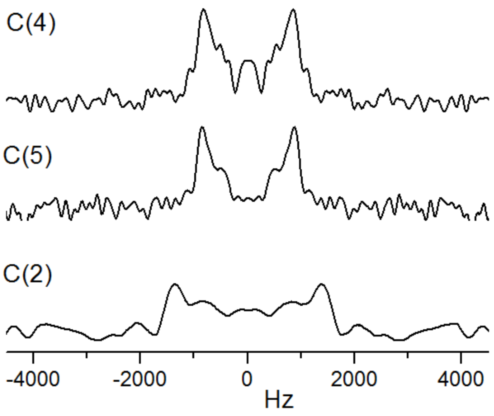

Figure S5. Cross-sections for the imidazolium carbons along the dipolar dimension from 2D APM-CP spectrum in $C_6(C_{12}im)_2Br_2$ smectic phase at 88 °C.

Table S2. Dipolar couplings in the imidazolium ring, estimated from APM-CP and PDLF experiments.

Carbon	$\|d_{CH}^{(APMCP)}\|$,[a)] Hz	$d_{CH}^{(PDLF)}$,[b)] Hz
C(2)	2029	−1901 (+1681)[c)]
C(4)	1189	+1168 (−1368)[c)]
C(5)	1195	−1146 (+946)[c)]

[a)] Calculated from APM-CP spectra.

[b)] Signs and magnitudes are inferred from Eq. (S1) by comparing $d_{CH}^{(PDLF)}$ to the values obtained from APM-CP spectra.

[c)] The value in the parenthesis is obtained assuming (incorrectly) the opposite sign of the coupling constant.

S6. ^{1}H NMR spectra

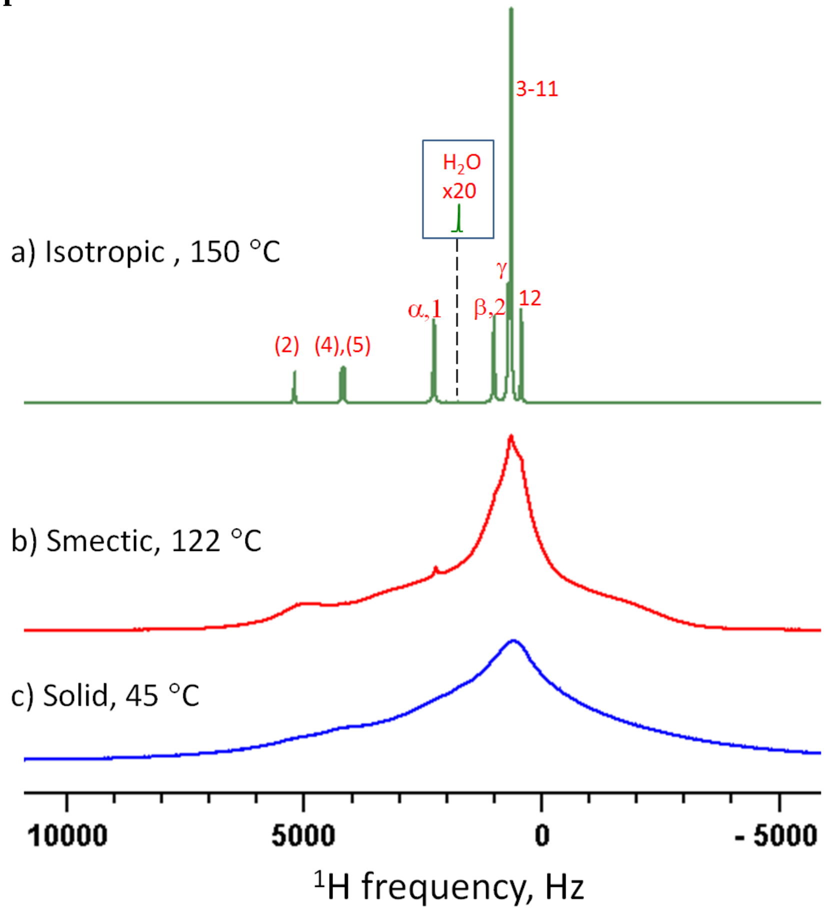

Figure S6. ^{1}H NMR spectra of the sample $C_6(C_{12}im)_2Br_2$ in different phases. The spectrum in the smectic phase was recorded in the aligned sample. The water peak is shown on a larger vertical scale in the insert in the spectrum (a).

The spectrum in the isotropic phase indicated the presence of 5 mol% water in the sample (Fig. S6a). This small amount of water was expected to have a negligible effect on the order and dynamics of ions[5-7].

References

1. Emsley, J. W. *et al.* A comparison of proton-detected C-13 local field experiments with deuterium NMR at natural abundance for studying liquid crystals. *Liq. Cryst.* **35**, 443-464 (2008).
2. Dvinskikh, S. V., Zimmermann, H., Maliniak, A. & Sandström, D. Measurements of Motionally Averaged Heteronuclear Dipolar Couplings in MAS NMR using R-type Recoupling. *J. Magn. Reson.* **168**, 194-201 (2004).
3. Dvinskikh, S. V. & Sandström, D. Frequency offset refocused PISEMA-type sequences. *J. Magn. Reson.* **175**, 163-169 (2005).
4. Dvinskikh, S. V., Castro, V. & Sandström, D. Probing Segmental Order in Lipid Bilayers at Variable Hydration Levels by Amplitude- and Phase-Modulated Cross-Polarization NMR. *Phys. Chem. Chem. Phys.* **7**, 3255-3257 (2005).
5. Goossens, K., Lava, K., Bielawski, C. W. & Binnemans, K. Ionic Liquid Crystals: Versatile Materials. *Chem. Rev.* **116**, 4643-4807 (2016).
6. Getsis, A. & Mudring, A. V. Imidazolium based ionic liquid crystals: structure, photophysical and thermal behaviour of [C(n)mim]Br·xH_2O (n=12, 14; x=0, 1). *Cryst. Res. Technol.* **43**, 1187-1196 (2008).
7. Puntus, L. N., Schenk, K. J. & Bunzli, J. C. G. Intense near-infrared luminescence of a mesomorphic ionic liquid doped with lanthanide beta-diketonate ternary complexes. *Eur. J. Inorg. Chem.*, 4739-4744 (2005).
